# Does Motor Symptoms Asymmetry Predict Motor Outcome of Subthalamic Deep Brain Stimulation in Parkinson's Disease Patients?

**DOI:** 10.3389/fnhum.2022.931858

**Published:** 2022-06-21

**Authors:** Francesco Bove, Francesco Cavallieri, Anna Castrioto, Sara Meoni, Emmanuelle Schmitt, Amélie Bichon, Eugénie Lhommée, Pierre Pélissier, Andrea Kistner, Eric Chevrier, Eric Seigneuret, Stephan Chabardès, Franco Valzania, Valerie Fraix, Elena Moro

**Affiliations:** ^1^Neurology Unit, Fondazione Policlinico Universitario A. Gemelli IRCCS, Rome, Italy; ^2^Neurology Unit, Department of Neuromotor and Rehabilitation, Azienda USL-IRCCS di Reggio Emilia, Reggio Emilia, Italy; ^3^Division of Neurology, Grenoble Institute of Neurosciences, Inserm U1216, CHU of Grenoble, Grenoble Alpes University, Grenoble, France; ^4^Division of Neurosurgery, CHU of Grenoble, Grenoble Alpes University, Grenoble, France

**Keywords:** deep brain stimulation, motor asymmetry, motor outcome, Parkinson's disease, predictors, subthalamic nucleus

## Abstract

**Background:**

In Parkinson's disease (PD), the side of motor symptoms onset may influence disease progression, with a faster motor symptom progression in patients with left side lateralization. Moreover, worse neuropsychological outcomes after subthalamic nucleus deep brain stimulation (STN-DBS) have been described in patients with predominantly left-sided motor symptoms. The objective of this study was to evaluate if the body side of motor symptoms onset may predict motor outcome of bilateral STN-DBS.

**Methods:**

This retrospective study included all consecutive PD patients treated with bilateral STN-DBS at Grenoble University Hospital from 1993 to 2015. Demographic, clinical and neuroimaging data were collected before (baseline condition) and 1 year after surgery (follow-up condition). The predictive factors of motor outcome at one-year follow-up, measured by the percentage change in the MDS-UPDRS-III score, were evaluated through univariate and multivariate linear regression analysis.

**Results:**

A total of 233 patients were included with one-year follow-up after surgery [143 males (61.40%); 121 (51.90 %) right body onset; 112 (48.10%) left body onset; mean age at surgery, 55.31 ± 8.44 years; mean disease duration, 11.61 ± 3.87]. Multivariate linear regression analysis showed that the left side of motor symptoms onset did not predict motor outcome (β = 0.093, 95% CI = −1.967 to 11.497, p = 0.164).

**Conclusions:**

In this retrospective study, the body side of motor symptoms onset did not significantly influence the one-year motor outcome in a large cohort of PD patients treated with bilateral STN-DBS.

## Introduction

Bilateral deep brain stimulation of the subthalamic nucleus (STN-DBS) is an effective treatment in patients with Parkinson's Disease (PD), allowing a significant improvement in cardinal motor symptoms of disease, motor complications (dyskinesias and fluctuations) and quality of life in the long-term follow-up (Bove et al., 2021). Several works have characterized the predictive factors of postoperative DBS outcomes to improve the selection phase and provide reliable prognostic information to patients (Cavallieri et al., 2021).

In the general PD population, the side of motor symptoms onset has been recently investigated as a potential predictor of disease severity or progression. In particular, left side lateralization has been related to greater motor and non-motor impairment (Cubo et al., 2020; Zhu et al., 2021) and faster motor symptom progression (Elkurd et al., 2021). Further, in PD patients with STN-DBS, worse neuropsychological outcomes have been described in patients with predominantly left-sided motor symptoms (Voruz et al., 2022). To date, it is unknown if left side lateralization might also be a predictor of worse motor outcome after STN-DBS.

In this study, we retrospectively assessed a cohort of PD patients who underwent STN-DBS to investigate if the side of motor symptoms onset represents a predictive factor of motor outcome.

## Materials and Methods

### Participants

This single-center retrospective study included all consecutive PD patients who underwent bilateral STN-DBS at Grenoble University Hospital (France) from 1993 to 2015. PD patients need to fulfill the following inclusion criteria to be included: age at surgery younger than 75 years; presence of disabling motor complications not well optimized with antiparkinsonian medications. Meanwhile, the presence of systemic comorbidities interfering with surgery, severe psychiatric disorders, dementia, severe brain atrophy or diffuse cerebral ischemic lesions on brain magnetic resonance imaging (MRI) were exclusion criteria. The surgical procedure has been previously described in detail (Limousin et al., 1995, 1998).

### Clinical Assessment

All patients were assessed before (baseline condition) and 1 year after bilateral STN-DBS surgery (follow-up condition). Demographic variables, PD characteristics, cognitive and neuroimaging data have been collected by reviewing medical records. The clinical evaluation was performed in accordance with the Core Assessment Program for Intracerebral Transplantations (CAPIT) (from 1993 to 1999) (Langston et al., 1992) and the Core Assessment Program for Surgical Interventional Therapies in Parkinson's Disease (CAPSIT-PD) protocol (from 2000 to 2015) (Defer et al., 1999). PD motor severity was quantified with the Hoehn and Yahr scale (H&Y) (Hoehn and Yahr, 1967) and the UPDRS part III score (from 1993 to 2010) (Fahn et al., 1987) or the Movement Disorder Society– sponsored revision of the UPDRS (MDS-UDPRS; from 2011) part III score (Goetz et al., 2008), as previously reported (Bove et al., 2020; Cavallieri et al., 2021). Preoperatively, each patient underwent an acute levodopa challenge to evaluate levodopa responsiveness. All patients were evaluated in the defined “OFF” condition (obtained after a 12-h antiparkinsonian medication withdrawal) and in the defined “ON” condition (obtained after 60 min and the administration of a 50% higher dose of the usual levodopa morning intake). One year after surgery, patients were reevaluated in the on-stimulation/off-medication condition. Moreover, an extensive neuropsychological assessment was performed before surgery including the Mattis Dementia Rating Scale (MDRS) (Marson et al., 1997), the frontal score (Pillon et al., 1995) and the Beck Depression Inventory-II (BDI-II) (Beck et al., 1996). The total amount of chronic antiparkinsonian medications was calculated as levodopa equivalent daily dose (LEDD) milligrams (mg) according to previously reported conversion factors (Tomlinson et al., 2010).

### Statistical Analysis

Continuous variables were expressed as mean (±SD) and median (range), while frequencies and percentage were calculated for categorical variables. The aim of the study was to evaluate if the side of motor symptoms onset represents a predictive factor of motor outcome 1 year after surgery. We selected as primary outcome measure the motor percentage change between the MDS-UPDRS part III score in the on-stimulation/off-medication condition 1 year after surgery and the preoperative score in the defined off condition.

To test if the variation of the outcome measure was statistically significant, we applied paired *t*-test or Wilcoxon signed rank test depending on the distribution of the continuous variables.

We selected different independent preoperative variables for regression modeling including: age at surgery; age of PD onset; sex; side of motor symptoms onset; PD duration at surgery; presence or absence of chronic vascular lesions on preoperative brain MRI; BDI-II score; Frontal Score; MATTIS score; MDS-UDPRS part III score and Hoehn and Yahr stage in the defined-off and defined-on condition; levodopa responsiveness; LEDD and motor phenotype (postural instability/gait disorders [PIGD]; tremor dominant, indeterminate) (Stebbins et al., 2013). We performed univariate and multivariate standard linear regression analyses to define if the side of motor symptoms onset could predict motor outcome at 1 year after surgery. We included in the standard multivariate model only significant variables from the univariate models. To rule out collinearity or absence of independence, pairwise correlations were checked among key covariates. Moreover, the two subgroups (right side vs. left side of motor symptoms onset) were compared to find significant differences in demographic and clinical variables. As regards to continuous and ordinal variables, the Kolmogorov–Smirnov test was used to verify the normal distribution. Considering that most of them was not normally distributed, the Mann-Whitney test was used. For categorical variables the chi-square independence test was applied. A *p* < 0.05 was considered significant and results were reported as standardized β coefficient followed by 95% confidence interval (CI) of β coefficient. Statistical analysis was performed using the IBM SPSS Statistics for Windows software version 20.0 (IBM, Armonk, NY, USA).

## Results

From a total of 546 consecutive PD patients treated with bilateral STN-DBS from 1993 to 2015 at the Movement Disorders Center of Grenoble University Hospital (France), we excluded from the analyses 313 patients because: incomplete medical records (*n* = 264); surgical complications responsible for persistent neurological sequelae (*n* = 18); other brain surgical procedures (*n* = 17); electrode misplacement (*n* = 14). Demographic and clinical characteristics of the remaining 233 patients included in the analysis are shown in Table 1. By comparing the two subgroups (right vs. left side of motor symptoms onset) no significant statistical differences in demographic and clinical variables were found, meaning that the two subgroups were homogenous, as reported in Table 1.

**Table 1:** Demographic and clinical characteristics of the PD patients included in the analysis (*n* = 233).

**Variable**	**No. (%); mean [SD]; median {range}**	**Patients with right side of motor symptoms onset**	**Patients with left side of motor symptoms onset**	***p* value**
Sex	143 (61.40%)/90 (38.60 %)	78 (64.50%)/43 (35.50 %)	65 (58.00%)/47 (42.00%)	0.383
Men/women				
Side of motor symptoms onset	121 (51.90%)/112 (48.10%)			
Right/Left				
Presence of chronic vascular lesions on preoperative brain MRI	173 (74.20%)/26 (11.20%)/34 (14.60%)	88 (72.70%)/16 (13.20 %)/ 17 (14.00 %)	85 (75.90%)/10 (8.90%)/17 (15.20%)	0.421
No/Yes/Missing data				
PD motor subtype	126 (54.10%)/33 (14.10%)/74 (31.80%)	67 (55.40%)/15 (12.40 %)/ 39 (32.20 %)	59 (52.70%)/18 (16.00%)/35 (31.30%)	0.811
PIGD/Indeterminate/TD				
Disease duration (years)	11.61 [± 3.87]; 12.00 {3.00–27.00}	11.40 [± 3.94]; 11.00 {3.00–27.00}	11.83 [± 3.79]; 12.00 {4.0–22.0}	0.276
Age at surgery (years)	55.31 [± 8.44]; 56.00 {29.00–74.00}	54.23 [± 8.84]; 55.00 {29.00–71.00}	56.48 [± 7.86]; 57.00 {31.00–74.00}	0.072
Age at PD onset (years)	43.89 [± 7.90]; 44.00 {19.00–62.00}	43.09 [± 8.01]; 44.00 {19.00–62.00}	44.76 [± 7.73]; 45.00 {24.00–62.00}	0.127
MDS-UPDRS part III defined-off condition	55.17 [± 17.04]; 53.70 {20.30–106.50}	54.62 [± 16.67]; 53.70 {20.30–105.40}	55.76 [± 17.50]; 52.60 {23.90–106.50}	0.724
MDS-UPDRS part III defined-on condition	17.95 [± 9.00]; 16.10 {3.50–62.30}	18.25 [± 8.57]; 15.50 {3.50–45.50}	17.63 [± 9.46]; 16.35 {3.50–62.30}	0.414
L-dopa responsiveness (% of improvement)	70.04 [± 14.43]; 71.87 {7.14–97.43}	68.64 [± 15.73]; 70.00 {7.14–97.43.}	71.54 [± 12.77]; 72.09 {30.00–97.22}	0.211
BDI-II	11.31 [± 7.33]; 9.00 {0.00–42.00}	11.93 [± 7.34]; 10.00 {0.00–42.00}	10.67 [± 7.30]; 9.00 {0.00–37.00}	0.145
MDRS	136.98 [± 6.01]; 139.00 {111.00–144.00}	136.93 [± 5.92]; 139.00 {116.00–144.00}	137.01 [± 6.14]; 138.00 {111.00–144.00}	0.905
Frontal score	39.01 [± 7.82]; 40.50 {17.60–50.00}	38.03 [± 8.38]; 39.30 {17.60–50.00}	40.18 [± 6.98]; 41.25 {23.40–50.00}	0.101
LEDD (mg)	1,356.85 [± 560.37]; 1,325.00 {265.00–4,200.00}	1,303.00 [± 540.08]; 1,265.00 {265.00–3,600.00}	1,414.17 [± 578.51]; 1,400.00 {320.00–4,200.00}	0.122

*BDI-II, Beck Depression Inventory-II; LEDD, L-dopa equivalent daily dose; MDRS, Mattis Dementia Rating Scale; MDS-UPDRS, Movement Disorder Society–sponsored revision of the UPDRS; MRI, magnetic resonance imaging; PD, Parkinson's disease; PIGD, dominant postural instability and gait disorder; SD, standard deviation; TD, tremor dominant*.

One year after surgery, the MDS-UPDRS part III scores in the on-stimulation/off-medication condition significantly decreased if compared with the preoperative medication-off condition (*p* < 0.001) (Table 2).

**Table 2:** Changes in the MDS-UPDRS part III score 1 year after STN-DBS surgery (*n* = 233).

**Variable**	**Baseline**		**1-year follow-up**
	**Defined off condition**	**Defined on condition**	**On-stimulation/off-medication condition**
**MDS-UPDRS part-III**			
mean [± SD];	55.17 [± 17.04];	17.95 [± 9.00];	16.69 [± 10.34];
median {range}	53.70 {20.30–106.50}	16.10 {3.50–62.30}	14.30 {2.30–61.40}

*MDS-UPDRS, Movement Disorder Society–sponsored revision of the UPDRS*.

The side of motor symptoms onset emerged as a possible predictor of motor outcome on univariate analysis together with age at surgery, age at PD onset, presence of chronic vascular lesions, MDRS, Frontal Score, the MDS-UPDRS part-III score in the defined-off condition, L-dopa responsiveness and tremor dominant phenotype. However, on the multivariate regression analysis, the side of motor symptoms onset was not confirmed as a predictor of STN-DBS motor outcome.

On the contrary, multivariate regression analysis confirmed that higher Frontal Score (β = 0.222, 95% CI = 0.202 to 1.253, *p* = 0.007) and higher MDS-UPDRS-III score in preoperative off-medication condition (β = 0.262, 95% CI = 0.193 to 0.597, *p* < 0.001) were significant predictors of a better motor outcome, while the presence of chronic vascular lesions on preoperative brain MRI (β = −0.158, 95% CI = −22.330 to −1.618, *p* = 0.024) was predictor of a worse outcome at 1 year after surgery. The results of the univariate and multivariate regression analyses are reported in Table 3.

**Table 3:** Preoperative predictive factors of motor outcome after STN-DBS (*n* = 233).

**Baseline variable**	**Univariate Analysis**			**Multivariate Analysis**		
	**Standardized β coefficient**	**95% CI**	***p* value**	**Standardized β coefficient**	**95% CI**	***p* value**
Left side of motor symptoms onset	0.150	1.10 to 14.24	0.022	0.093	−1.967 to 11.497	0.164
Age at surgery	−0.268	−1.195 to −0.436	<0.001	0.053	−0.664 to 0.986	0.701
Age at PD onset	−0.296	−1.362 to −0.558	<0.001	−0.148	−1.347 to 0.389	0.277
Presence of chronic vascular lesions on preoperative brain MRI	−0.301	−33.055 to −12.693	<0.001	−0.158	−22.330 to −1.618	0.024
MDRS	0.256	0.538 to 1.650	<0.001	0.087	−0.281 to 1.020	0.264
Frontal Score	0.361	0.751 to 1.615	<0.001	0.222	0.202 to 1.253	0.007
MDS-UPDRS part-III off-medication	0.298	0.263 to 0.635	<0.001	0.262	0.193 to 0.597	<0.001
L-dopa responsiveness	0.302	0.317 to 0.757	<0.001	0.133	−0.003 to 0.476	0.053
TD phenotype	0.173	1.268 to 8.545	0.008	0.103	−0.765 to 6.611	0.119

*MDRS, Mattis Dementia Rating Scale; MDS-UPDRS, Movement Disorder Society–sponsored revision of the UPDRS; MRI, magnetic resonance imaging; PD, Parkinson's disease; TD, tremor dominant*.

## Discussion

In this large cohort of PD patients, the side of motor symptoms onset did not predict the motor outcome of STN-DBS at 1 year after surgery. Although in the general PD population left side lateralization has been related to worse motor outcomes (Cubo et al., 2020; Elkurd et al., 2021; Fiorenzato et al., 2021), DBS was equally effective in restoring motor symptoms regardless of motor side lateralization. Further, the good motor response to STN-DBS of both the groups of left- and right-dominant side patients was not in contrast with previous findings of different neuropsychological outcomes according to the motor side lateralization (Voruz et al., 2022). As a matter of the facts, motor and non-motor outcomes of STN-DBS are not strictly related, as they likely rely on different factors and different neurobiological basis (Kurtis et al., 2017).

In this well-selected population (with a good preoperative responsiveness to levodopa), the main predictors of DBS motor outcome were the Frontal Score, the MDS-UPDRS-III score in the preoperative off-medication condition, and the presence of chronic vascular lesions on preoperative brain MRI, as recently reported by our group (Cavallieri et al., 2021). The side of motor symptoms onset did not interact with these factors and did not add a risk of poor motor response. It is interesting to note that the two subgroups were homogenous for both clinical and demographic variables, thus allowing to exclude the possible bias related to inter-group differences.

The main limitation of the study is the unavailability of information about the dominant hemisphere of the patients. In fact, it has been previously postulated that dominant hemisphere would have more efficient motor networks with greater neural reserve, and this may influence motor phenotype and disease progression (Ham et al., 2015). Another limitation to be considered is the retrospective nature of the study.

## Conclusions

The side of motor symptoms onset does not influence the motor outcome of PD patients with one-year STN-DBS. Other predictive factors should be considered before surgery. Our findings are relevant to the clinicians in the preoperative selection phase and to properly inform the patients.

## Data Availability Statement

The raw data supporting the conclusions of this article will be made available by the authors, without undue reservation.

## Ethics Statement

The studies involving human participants were reviewed and approved by Grenoble CHU Research Center Authority. Written informed consent for participation was not required for this study in accordance with the national legislation and the institutional requirements.

## Author Contributions

FB and FC conceived the study and were responsible for data analysis and writing the first draft of the manuscript. AC, SM, ESc, AB, EL, AK, PP, EC, ESe, SC, and VF performed the clinical evaluations of the patients and collected the data. FV was responsible for data analysis and writing the first draft of the manuscript. EM conceived the study and revised the manuscript for intellectual content. All authors read and approved the final manuscript.

## Conflict of Interest

FC received personal fees from Zambon outside the submitted work. AC received research grants from France Parkinson Association and Medtronic. SM has received grant support from Medtronic. SC received grants and personal fees from Medtronic and Boston Scientific. VF received honoraria from AbbVie and Medtronic for consulting services and lecturing. EM has received honoraria from Abbott, Medtronic, Kyowa and Newronika for consulting and lecturing and received an educational grant from Boston Scientific. The remaining authors declare that the research was conducted in the absence of any commercial or financial relationships that could be construed as a potential conflict of interest.

## Publisher's Note

All claims expressed in this article are solely those of the authors and do not necessarily represent those of their affiliated organizations, or those of the publisher, the editors and the reviewers. Any product that may be evaluated in this article, or claim that may be made by its manufacturer, is not guaranteed or endorsed by the publisher.
